# Nanoporous Microsphere Assembly of Iodine-Functionalised Silver Nanoparticles as a Novel Mini-Substrate for Enriching and Sensing

**DOI:** 10.1038/srep46640

**Published:** 2017-04-19

**Authors:** X. -L. Wu, H. Wu, Z.-M. Wang, H. Aizawa, J. Guo, Y.-H. Chu

**Affiliations:** 1 College of Architecture and Environment, Sichuan University, Chengdu 610065, Sichuan, China; 2 Environmental Management Research Institute, National Institute of Advanced Industrial Science and Technology, 16-1 Onogawa, Tsukuba, Ibaraki 305-8569, Japan

## Abstract

Herein, debris particulates of nanoporous silver (np-Ag) were synthesised by a dealloying method, and their integration behaviour and surface-enhanced Raman scattering (SERS) properties during iodine functionalisation were examined. It was found that the dealloyed np-Ag debris particulates gradually assembled to form rigid nanoporous microspheres comprising Ag nano-ligaments due to mechanical collisions during iodine treatment. High-resolution transmission electron microscopy and X-ray photoelectron microscopy clearly showed the iodide surface of np-Ag, which was dotted with iodine or iodide ‘nanoislands’. The exceptional, and unexpected, integration and surface structures result in a highly enhanced localised surface plasmon resonance. Furthermore, the robust nanoporous microspheres can be employed individually as as-produced miniaturised electrodes to electrically enrich target molecules at parts-per-trillion levels, so as to achieve charge selectivity and superior detectability compared with the ordinary SERS effect.

Owing to their high active surface areas and excellent electrical and thermal conductivities, nanoporous metals have attracted a great deal of attention for potential applications in catalysis and electrochemical catalysis, sensing and electronic devices^1^,^2^,^3^,^4^,^5^,^6^. Nanoporous silver (np-Ag) is one type of nanoporous metal with lower price compared with gold and platinum but better chemical stability compared with base metals such as copper and nickel. Among several fabrication approaches for nanoporous metals^7^,^8^, dealloying is a process involving the corrosion of an alloy, which can give a monolithic sponge-like nanoporous structure made from ligament linkages of metallic nanoparticles such as silver nanoparticles (Ag NPs). During the dealloying process, chemically or electrochemically active elements of an alloy are selectively dissolved at the interface, and the remaining more noble element is chemically driven to aggregate into a two-dimensional cluster by an intrinsic phase separation process (spinodal decomposition), finally interconnecting to form a continuous nanoporous framework^9^,^10^.

The dealloyed nanoporous framework of Ag NPs (np-Ag-NPs) has been recently synthesised and applied in high-efficiency catalysis and electrochemical catalysis^11^ and as sensing substrates for surface-enhanced Raman scattering (SERS)^12^,^13^. As a powerful and non-destructive technique, the SERS effect is known to be dependent on the substrate structure and configuration because the amplification of light resulting in electromagnetic enhancement occurs preferentially in the gaps, crevices, or sharp features of plasmonic materials at the nanoscale. Silver has localised surface plasmon resonances in most of the visible and near-infrared wavelength range (400–1000 nm). Up to date, Ag NPs with various shapes such as nanocube, nanopyramid, nanorod, nanowire^14^ and various configuration structures^15^,^16^,^17^ have been synthesised, and their surface modifications (for example, using iodine (I_2_) treatment^18^,^19^) have been studied to achieve increased SERS enhancement. Although it is beneficial for adsorption concentration or enrichment of trace target molecules, the fabrication of porous frameworks with integrity using plasmonic nanoparticles has not been sufficiently explored so far. In this regard, the np-Ag-NP monolith is a hopeful candidate structure. However, the np-Ag-NP monolith is usually weak in strength because of its loose porous sponge structure. It is easily broken to random-sized debris particulates of small np-Ag-NP aggregates by exerting mechanical pressure or external shocking. It is also readily collapsed even during the formation process, depending on synthesis conditions (acid concentration, contact time, etc.). This fragile property reduces their durability and convenience in use. On the other hand, on-site field application using small and portable Raman spectrometers is another challenging trend for SERS detection which requires advent and evolution of rigid and efficient miniature substrates as small as the size for use, for example, in a smartphone^15^.

Herein, debris particulates of np-Ag were functionalised by iodine in liquid ethanol. Iodine is known to have a strong chemical affinity for silver and has been exploited to enable silver with tuned surface dielectric properties, improved conductive contacts and enhanced SERS sensitivity^18^,^19^,^20^,^21^. Our motives in the first place are to attain a nanoporous Ag substrate with a higher Raman enhancement effect. However, we happen to find that the liquid functionalisation course is an effective way to facilitate the self-assembly of Ag NPs into a robust, close-contacted but void-spaced spherical aggregate of micrometre size. The novel np-Ag-NP microsphere meets the requirement for an effective as-produced mini-substrate with a strong SERS enhancement effect. At the same time, the individual microspheres can serve as rigid microelectrodes for portable use, whose nanoporosity and conductivity allow for electrical enrichment of trace pollutant molecules with charge selectivity.

## Results and Discussion

**Figure 1:**
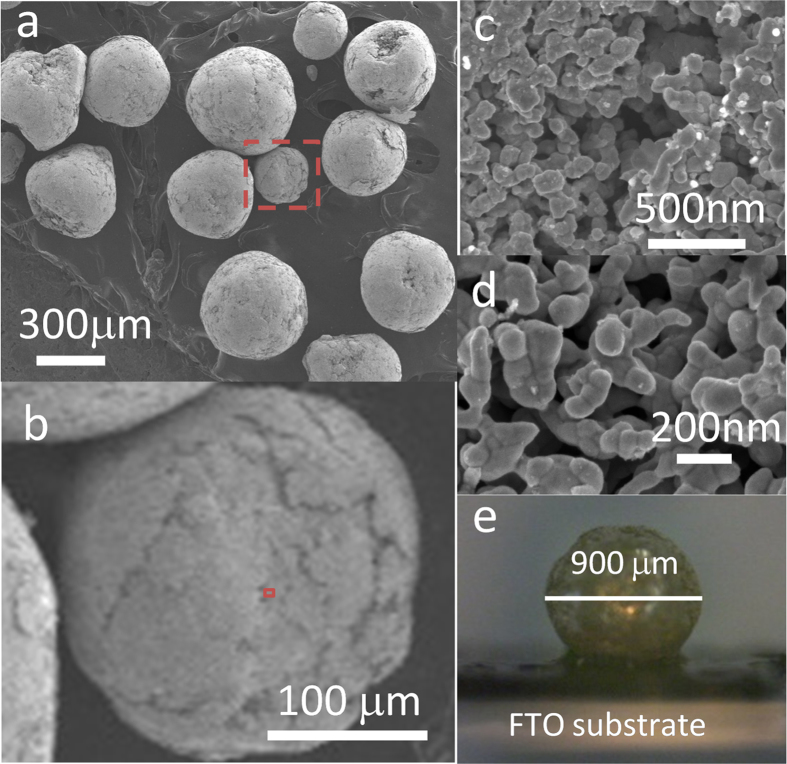
SEM and optical microscope (OM) images. SEM images of (**a,b**) np-Ag-NP microspheres and (**c,d**) the expanded surface (the red square part in **b**), and (**e**) OM image of the microsphere glued on a fluorine-doped tin oxide substrate using a double-sided conductive carbon tape.

**Figure 2:**
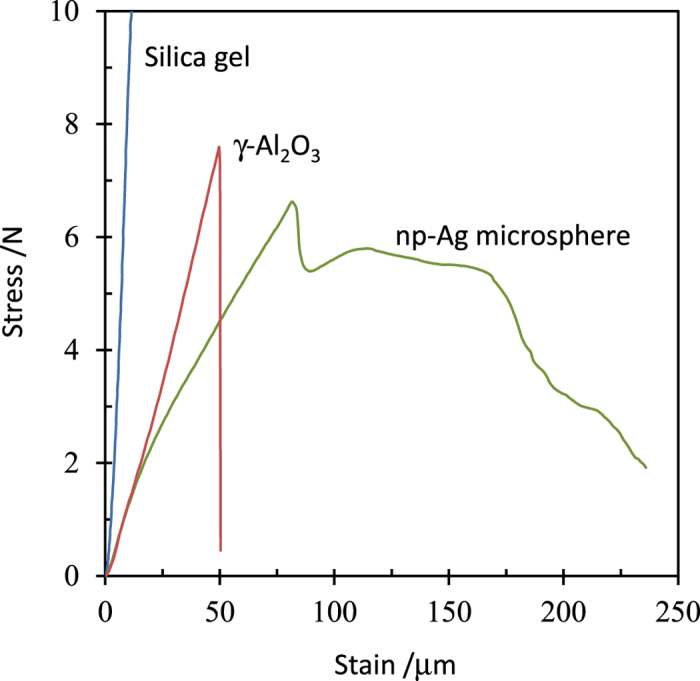
Mechanical properties. Stress–strain curve of the np-Ag microsphere compared with those of a ϕ4 mm γ-Al_2_O_3_ pellet and a silica gel pellet. The np-Ag microsphere and γ-Al_2_O_3_ were measured by the Shimadzu MTS-I micro strain tester and silica was measured by the Shimadzu EZ-LX test machine. The stress is compared based on the force because of the spherical shape.

The np-Ag-NP debris particulates used to obtain the microsphere assemblies were synthesised using a chemical dealloying method^22^,^23^. To make the alloy precursor, a hydraulically pressed ϕ1 cm round disc of Ag and Al powder mixture was sintered, annealed and quenched in ice water (Supplementary Information). During the process, the crystal phase of the alloy experienced changes from a mixed phase of α-Al(Ag) and various intermetallic compounds (Ag_2_Al 2 H, AlAg_3_ 20P) to a near 100% pure α-Al(Ag)-type solid solution phase (Supplementary Fig. S1a–c)^11^,^22^. The polished silver-colour alloy was then subjected to dealloying in hydrochloride acid to selectively dissolve Al, from which np-Ag-NP monolith or its cracked/collapsed particulates were obtained under harsh conditions (higher concentration and longer time). The np-Ag-NP monolith is a loose sponge structure and is easily crumpled by pricking with tweezers or even by sonication to random-sized smaller debris particulates. The debris particulates retain the nanoporous interlinkage structure of Ag nano-ligaments (Supplementary Fig. S2a) which are short wormlike 1D aggregations of Ag NPs with an average diameter of around 75–80 nm (Supplementary Fig. S3). The np-Ag-NP debris was then immersed in an ethanol solution of iodine for functionalisation under continuous magnetic stirring. Prior to the treatment, the solution was transformed by sonication to a dispersion of smaller Ag NPs that can keep floating in the solution for a long time, characteristic of their nanoporous properties. Upon proceeding with I_2_ functionalisation, small Ag NPs were found to gradually gather together (Supplementary Fig. S2b) and assemble to a microsphere-like aggregate with a diameter of several hundred micrometres after 24 h (Fig. 1a,b). The microspheres show a smooth surface comprising both close-packed (upper-left, lower-right areas of Fig. 1c) and loose-packed (Fig. 1d) Ag NPs compared with the precursor debris particulates. They are robust enough to be picked up by tweezers to glue onto or remove from a conductive substrate without destruction (Fig. 1e). They still remain nanoporous with void openings (ligament spacings) of several tens to two hundred nanometres and a porosity of 40–45% from geometric estimations. Figure 2 shows the stress–strain curve of an np-Ag microsphere in comparison with those of a ϕ4 mm γ-alumina pellet and a ϕ4.6 mm silica gel pellet. The peculiar porous property of the microsphere leads to a complicated stress–strain behaviour which demonstrates that a secondary aggregate structure was constructed after the initial structural fracture at ~6.6 N, which resists against almost the same stress from 88 to 160 μm, revealing a greater compressibility of np-Ag microspheres compared with the brittle alumina. The several Newton force for the structural fracture of the np-Ag microsphere is comparable with that of the γ-alumina pellet. Thus, the np-Ag microsphere is not as hard as diamond or silica gel, but strong enough for picking up and transferring in most portable uses.

**Figure 3:**
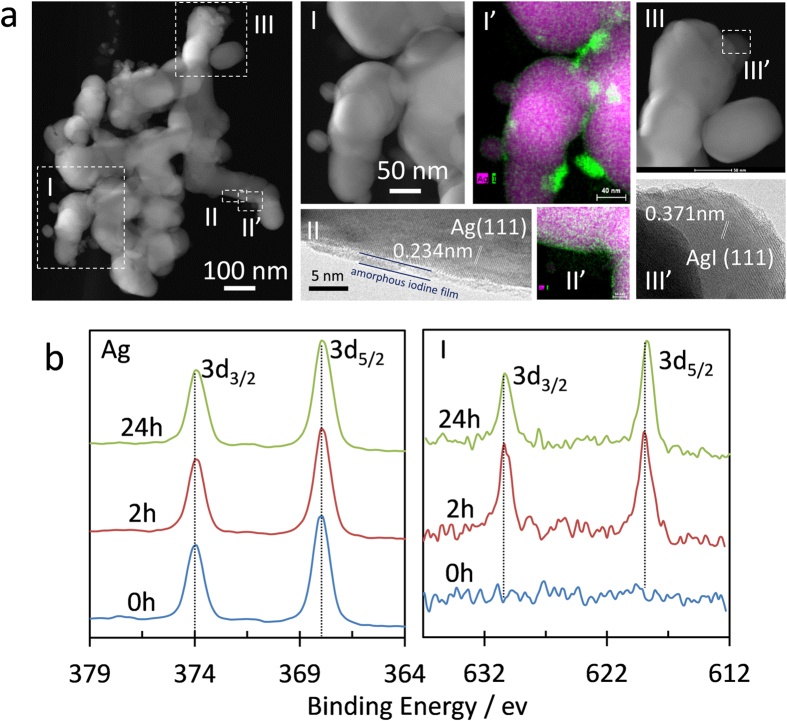
TEM images and XPS results. (**a**) HAADF-STEM, I (green)- and Ag (pink)-elemental mapping and HRTEM images of the np-Ag-NP debris functionalised with I_2_ for 2 h, and (**b**) Ag 3d and I 3d XPS spectra of np-Ag-NPs functionalised with I_2_ for different times.

Regarding the formation mechanism of the np-Ag-NP microsphere, we consider that the mechanical collision-induced twisting/entangling of ligaments in np-Ag-NP debris particulates driven by magnetic stirring plays an important role. In addition, surface modification by I_2_ functionalisation can accelerate the compacting of Ag NPs, because this favours lowering or compensation of the surface energy of the polar or coarsened surface during the reaction with I_2_. It was reported that iodine can erode the surface of silver to form a AgI dielectric film or nano-sized islands of silver on the silver film^18^,^19^,^20^,^21^. Indeed, we observed a lot of separated silver nanoparticle deposits on the surface of the Ag ligaments after iodine treatment (Fig. 1c,d). From the high-angle annular dark-field scanning transmission electron microscopy (HADDF STEM), high-resolution transmission electron microscopy (HRTEM) and energy dispersive X-ray mapping images (Fig. 3a), iodine was found to either distribute around the small nanoparticles that are attached on the surface of the Ag ligament or directly deposit on the surface of the Ag nano-ligaments after 2 h treatment (I, I′, II, II′ in Fig. 3a). Besides Ag NPs, AgI nanoparticle islands with (111) lattice fringe were also identified on the Ag ligament surface (III and III’ in Fig. 3a). Furthermore, although the X-ray photoelectron spectroscopic (XPS) results (Ag 3d at ~368.1 and ~374.1 eV) (Fig. 3b) together with the X-ray diffraction (XRD) results (Supplementary Fig. S1e–g) confirmed that the major structure of the treated np-Ag-NP samples was still face-centred-cubic (fcc) Ag^0 ^24, iodine was found to remain in the 24 h-treated microsphere sample. The position of the I 3d_5/2_ peak (~619 eV with a spin-orbital splitting of ~11.5 eV) of the I_2_-treated samples is close to that of iodide or molecular iodine chemisorbed on Ag (Fig. 3b)^24^, which slightly shifts downward for the microsphere sample, indicating the intensifying of iodination. The above facts thus ascertain that iodine does erode the silver surface.

To further clarify the role of I_2_ in the microsphere formation, we treated the np-Ag-NP debris particulates in pure ethanol without iodine. It was found that a microsphere assembly was also formed in this case; however, the forming speed is slower, and both the yield (~61% on average) and size (mainly ~200 nm) of the microspheres are lower than those of the case using I_2_ (>95% and mainly ~500 nm, respectively), when 10 mg of debris particulates were used in both cases. In addition, since mechanical collision of np-Ag-NPs in I_2_ solution results in a smooth microsphere surface assembled from void (loose-packed areas)-interspersed close-packed Ag nano-ligaments (Fig. 1c), it is possible that the Ag ligaments may experience physical changes during the process. This is further evidenced by a detailed counting of the ligament size distribution, which showed that the ligament size in the close-packed area of the I_2_-functionalised microspheres distributes downward compared with that in the loose-packed (porous) part, leading to a smaller total average size than that of the debris precursor (Supplementary Fig. S3). Hence, reaction with iodine ‘scrapes’ out the surface of the silver ligament to form separated Ag or AgI nanoparticles and, as a result, promotes their compacting into a microsphere assembly.

**Figure 4:**
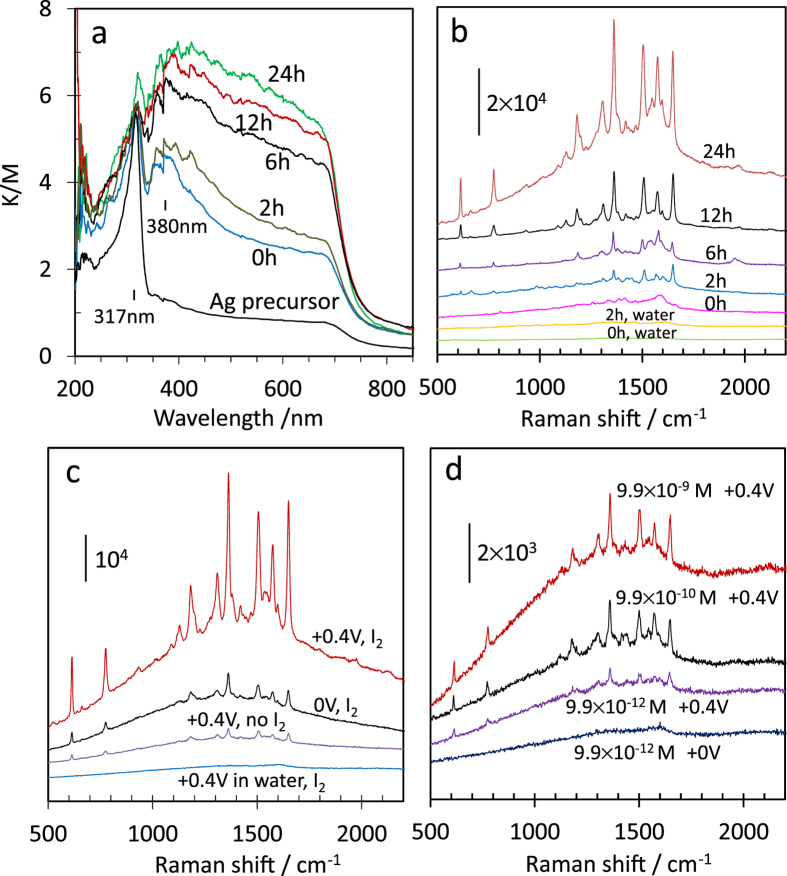
UV-vis DRS of the samples and SERS Raman spectra of R6G at different conditions. (**a**) UV-vis DRS of np-Ag-NPs functionalised for different times, and SERS spectra of R6G on (**b**) np-Ag-NPs functionalised for different times, where R6G was adsorbed by evaporation to dryness from 50 μL of a 9.9  ×  10^−8^ M solution, (**c**) the microspheres formed by 24 h treatment with (I_2_) and without (no I_2_) iodine, where R6G was electrically (+0.4 V) or non-electrically (0 V) adsorbed from 15 mL of a 9.9 × 10^−8^ M solution for 1 min, and (**d**) the I_2_-functionalised microsphere after electrical enrichment of R6G from lower concentration solutions. The bottom two plots in (**b**) and the bottom plot in (**c**) are controls using distilled water only. The values of bias voltage or R6G concentration are shown in (**c**) and (**d**).

The localised surface plasmon resonance (LPSR) properties of the np-Ag-NPs can be characterised by UV-vis diffuse reflectance spectroscopy (UV-vis DRS). As shown in Fig. 4a, all np-Ag-NP samples before and after I_2_ functionalisation exhibit an absorption at 317 nm, which corresponds to the bulk plasma edge of silver^25^. Another absorption peak at 380 nm for the debris precursor (0 h) is attributed to the plasmonic resonance by nanostructured Ag ligament with an average size of around 75 nm (Supplementary Fig. S3). The asymmetry of the peak is related to the elongated shape of Ag ligaments^25^,^26^,^27^. With I_2_ functionalisation, the peak at 380 nm red-shifts with increasing absorbance gradually. The similar behaviour resulted from cluster formation of Ag nanoparticles in previous reports^26^, which is consistent with the tendency of compacting and close-contacting of Ag nano-ligaments in this work.

It has been reported that np-Ag-NP sponges contain a lot of hot spots that cause strong SERS sensitivity^12^,^13^. The I_2_-functionalised np-Ag-NP microsphere can thus be expected to have a further improved LPSR property because its surface contains more additional hot spots, such as the interstice or groove sites between the compacted Ag nano-ligaments, those between the ligament surface and the deposited Ag nanoislands, and some parts of the roughened surface of the nano-ligament itself, as a consequence of I_2_ erosion. The enhancement can be evidenced by the SERS signals of rhodamine 6 G (R6G) dye on the materials. Figures 4b–d show the SERS spectra measured intentionally using a very low laser power (0.15 mW) to highlight the higher detection sensitivity. As shown in Fig. 4b, only a negligible signal of R6G was observed on the debris precursor (note that the similar material showed significant signals at the same concentration level under the measurement conditions of reported literature^13^), whereas the SERS signal increases progressively with I_2_ treatment, reaching a very high level after 24 h. It is rather interesting that the robust np-Ag-NP built-in microsphere can be easily stuck on the top of a conductive substrate (Fig. 1e) and used as a micro-size working electrode to electrically enrich/adsorb target molecules. As shown in Fig. 4c, the SERS peak intensity of R6G on the I_2_-functionalised microsphere was increased by ~6.5 times by applying a positive bias voltage compared with the condition without bias voltage. This implies that the positively charged np-Ag-NP microsphere is very effective at quickly attracting and enriching the negatively charged R6G anions in solution within as little as 1 min. The signal intensity is also ~16 times higher than that of the non-iodine-treated counterpart even under + 0.4 V bias voltage, signifying the favoured role of I_2_ functionalisation in the enhancement of SERS sensitivity. Figure 4d shows that the I_2_-functionalised microsphere is sensitive to R6G of as low as 9.9 × 10^−12^ M with the aid of a positive bias voltage, even though it is almost undetectable without applying a bias voltage. It is remarkable that 9.9 × 10^−12^ M equivalent to 4.7 parts-per-trillion (ppt) is an extremely low concentration. The detectability resulting from a several-fold or several-tenfold increase in the enhancement factor at such an ultra-low concentration range can provide a critical advantage in the direct detection of organic pollutants or biomolecules that are environmentally, biochemically, or medically essential at sub-ppb levels. Therefore, without employing traditional concentrating procedures such as solid-phase extraction, the I_2_-functionalised np-Ag-NP microsphere can make excellent use of the electrical enriching force to achieve charge-selective ultra-low concentration detectability by a promoted SERS effect.

In conclusion, the facile mechanical collision approach toward np-Ag-NP debris opens a new door to novel micro-sized and robust-but-nanoporous spherical assemblies of Ag NPs. The microsphere material is an as-produced mini-substrate applicable to charge-selective and high-performance SERS sensing with the aid of electrical enrichment. The material can also be used as a prospective miniaturised reaction field for other applications such as micro devices and micro direct methanol fuel cells (μ-DMFC)^7^.

## Materials and Methods

### Synthesis of Ag-Al alloy precursor

Powders of 99.7% purity Ag and 99.99% purity Al were mixed in agate mortar at a weight ratio of 20:80 and the mixture was put into a *ϕ*1 cm dice to be hydraulically pressed under 60 MPa to a round cake of different thicknesses depending on the amount employed. The round mixture cake was then sintered at 873 K in nitrogen stream (250 ml/min) for 2 h. After cooling down to room temperature, the round cake was further annealed at 819 K in nitrogen stream (250 ml/min) for 12 h, following which a quick quenching process was applied to rapidly chill the annealed cake from 819 K to ~274 K using a water/ice bath.

### Synthesis of nanoporous silver nanoparticles (np-Ag-NPs) monolith or debris

Chemical dealloying method using hydrochloride acid (HCl) as the erosion species was employed to obtain np-Ag-NPs monolith or debris. After polishing with sandpaper and rasp to remove surface rust, the annealed cake was first immersed in 5 wt% HCl solution to allow leaching of Al for 30 min. Subsequently, the cake was moved to a 1 wt% HCl solution and the dealloying process was continued until no gas bubbles were released from the solution. The annealed cake usually transformed to np-Ag-NPs monolith after these procedures whereas it occasionally formed cracks during the dealloying process and finally collapsed to smaller pieces of np-Ag-NPs debris, especially when smaller total amount of metal mixture was employed. The np-Ag-NPs monolith or debris was thoroughly washed with distilled water, dried at 333 K in vacuum for 2 h, and reserved in a vacuum desiccator.

### Iodine functionalization of np-Ag-NPs

0.01~0.1 g np-Ag-NPs debris either as-synthesized or easily prepared by pricking the np-Ag-NPs monolith with tweezers was immersed in 5~10 ml ethanol. After sonication for 2 min, the solution became a uniform dispersion in which Ag NPs can suspend for a long time. Then a 0.1 mM I_2_ ethanol solution was slowly dropped under magnetic stirring. After adding over 7.9~15.8 ml of I_2_ ethanol solution in 15 min, the solution was kept stirring continuously for 2, 6, 12, or 24 h to assure further reaction. Finally, the I_2_ functionalized np-Ag samples were collected by centrifugation, sufficient washing with distilled water, and dried at 333 K in vacuum oven for 2 h.

### Characterization method

The XRD patterns of the materials were recorded on a Rigaku SmartLab diffractometer with K*α* irradiation (λ = 0.15406 nm) at 30 mA and 40 kV.

The images of field-emission scanning electron microscope (FE-SEM) were obtained by a Hitachi S-4700 type apparatus and also a JEOL JSM-6010LA type apparatus at an acceleration voltage of 2~5 keV and a working distance of 4 cm. Optical microscopic pictures were recorded on a Keyence VHX-500 type digital microscope. Transmission electron microscopic (TEM) observations and elemental mapping analysis were carried out by a FEI Tecnai Osiris type apparatus equipped with a high-angle annular dark field scanning TEM mode. The electron acceleration voltage was 200 keV.

The mechanical properties of the microsphere sample were evaluated by a Shimadzu EZ-LX type high resolution test machine (load cell 5 kN, test force measurement precision of ± 0.5% in the range >10 N, displacement measurement precision of ± 10 μm, test speed 1 mm·min^−1^) and a Shimadzu MST-I type high precision micro strain tester (load cell 50 N, test force measurement precision of ± 1% in the range >0.2 N, displacement measurement precision of ± 0.2 μm up to 5 mm, test speed 0.2 mm·min^−1^). Those of a ϕ 4 mm silica gel pellet (Tokai Kagaku Co. Ltd.-made desiccant) and a ϕ 4.6 mm sol-gel γ-alumina pellet (Mizusawa-Chem Co. Ltd.-made GB13 type catalyst support) were also measured for comparisons. Absolute force was compared in the stress-strain curves because of the spherical shapes.

The XPS spectra were obtained on an ULVAC-PHI 5000 Versa Probe type instrument with an Al Kα irradiation (15 kV, 25 W, beam diameter: 0.1 mm) and a neutralizer using argon ions and electron beams. The ultraviolet-visible diffuse reflectance spectra (UV-vis DRS) were recorded by a JASCO V-650 type UV-Vis spectrometer with a DRS attachment using barium sulfate as the reference.

### SERS experiments

R6G was deposited on the surface of the np-Ag-NPs materials either by the evaporation-to-dryness method or by solution adsorption method with or without exertion of electrical field. For the evaporation-to-dryness method, 9.5 mg material powder was loaded on the surface of a slide glass onto which 50 μL of 9.9 × 10^−8^ M R6G solution was dripped. R6G was then deposited on the materials by drying out the water solvent at ambient temperature and by further drying at 323 K for 40 min. For the solution adsorption method, an ng-Ag microsphere was immobilized on fluorine doped tin oxide (FTO)-coated glass substrate by a double-sided conductive carbon tape which was then immersed in 15 mL R6G solution of various concentrations (9.9 × 10^−12^, 9.9 × 10^−10^, 9.9 × 10^−9^, and 9.9 × 10^−8^ M). After electrical or non-electrical adsorption in solution for 1 min, the ng-Ag microsphere-loaded FTO substrate was removed from the solution and dried at 323 K in an air oven for 1 h. Electric field was exerted by a CH instrument CHI Model 760AC type electrochemical station. A 3-electrode system was employed, which uses Pt wire as the counter electrode, Ag/AgCl aq. as the reference electrode, and the ng-Ag microsphere-loaded FTO substrate as the working electrode. The R6G solution without other additional electrolytes was employed. RAMAN measurement was carried on by a Renishaw inVia Reflex type confocal Raman microscope system which uses an argon ion laser (514.5 nm, 15 W) as the excitation source and a peltier-cooled charge-coupled device (CCD) as detector. The laser beam at a laser power of 0.15 W (1% of the source power) was focused on the samples through a 50x microscope objective and spectra of more than 10 positions were measured.

## Additional Information

**How to cite this article:** Wu, X.-L. *et al*. Nanoporous Microsphere Assembly of Iodine-Functionalised Silver Nanoparticles as a Novel Mini-Substrate for Enriching and Sensing. *Sci. Rep.* **7**, 46640; doi: 10.1038/srep46640 (2017).

**Publisher's note:** Springer Nature remains neutral with regard to jurisdictional claims in published maps and institutional affiliations.

## Supplementary Material

Supplementary Information
