# Supplementary material for: Nanoporous Microsphere Assembly of Iodine-Functionalised Silver Nanoparticles as a Novel Mini-Substrate for Enriching and Sensing
Source: Sci Rep. 2017 Apr 19;7:46640. doi: 10.1038/srep46640 (PMC5395945; doi:10.1038/srep46640)
Supplement: Supplementary Information [file srep46640-s1.pdf]

Supplementary information

Nanoporous Microsphere Assembly of Iodine-Functionalised Silver Nanoparticles as a Novel Mini-Substrate for Enriching and Sensing

X. –L. Wu,[§, ‡] H. Wu,[‡] Z. –M. Wang,[*‡] H. Aizawa,[‡] J. Guo,[§] Y. –H. Chu[§]

[§]College of Architecture and Environment, Sichuan University, No.24 South Section 1, Yihuan Road, Chengdu 610065, Sichuan, China

[‡]Environmental Management Research Institute, National Institute of Advanced Industrial Science and Technology, 16-1 Onogawa, Tsukuba, Ibaraki 305-8569, Japan

[*]zm-wang@aist.go.jp (Corresponding author)

1. Supporting Figures

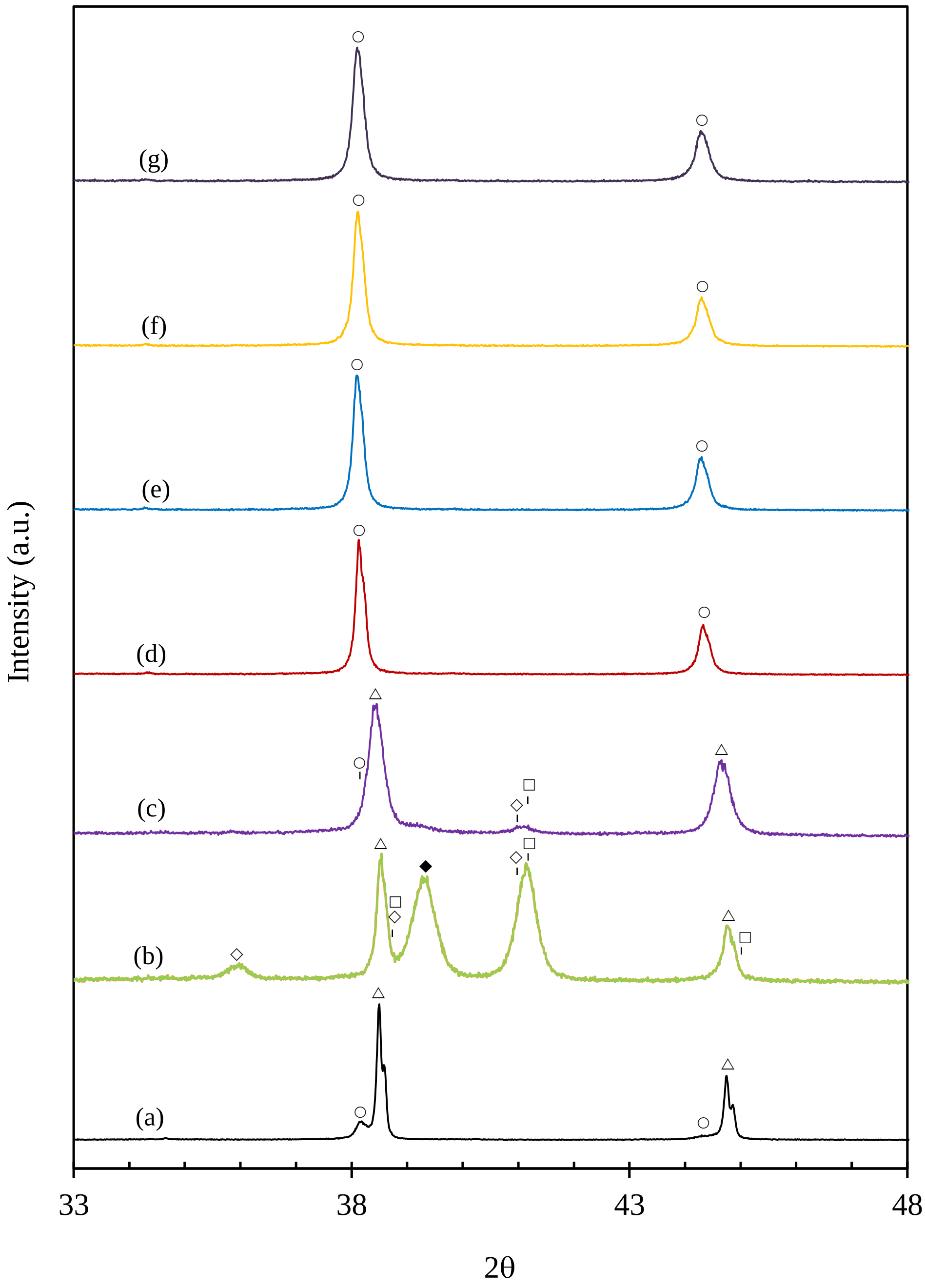

Figure S1. XRD patterns of (a) a ϕ1 cm round-shaped plate of Ag and Al power mixture formed under hydraulic press at 60 MPa, Ag-Al alloy obtained by (b) sintering at 873 K and (c) subsequent annealing and quenching at 819 K in nitrogen, (d) the dealloyed np-Ag-NPs debris, and those after iodine functionalization for (e) 2, (f) 6, and (g) 12 h.

○, Ag; △ , Al/α-Al(Ag); ◇, Ag_2Al; □, Ag_3Al; ◆, $AlAg_3$

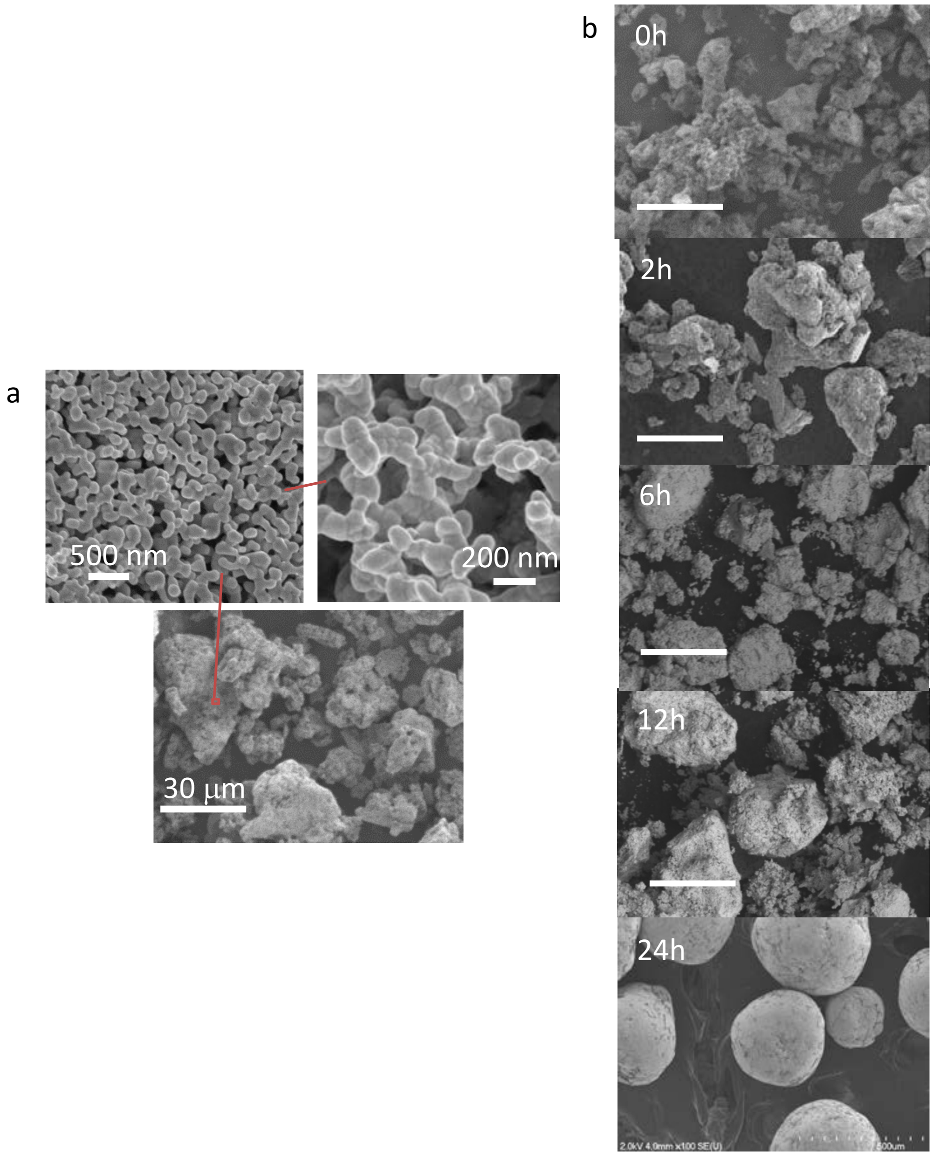

Figure S2. FE-SEM pictures of (a) the dealloyed np-Ag-NPs debris and (b) the debris after iodine functionalization at different times. Scale bars are 30 μm otherwise depicted.

.

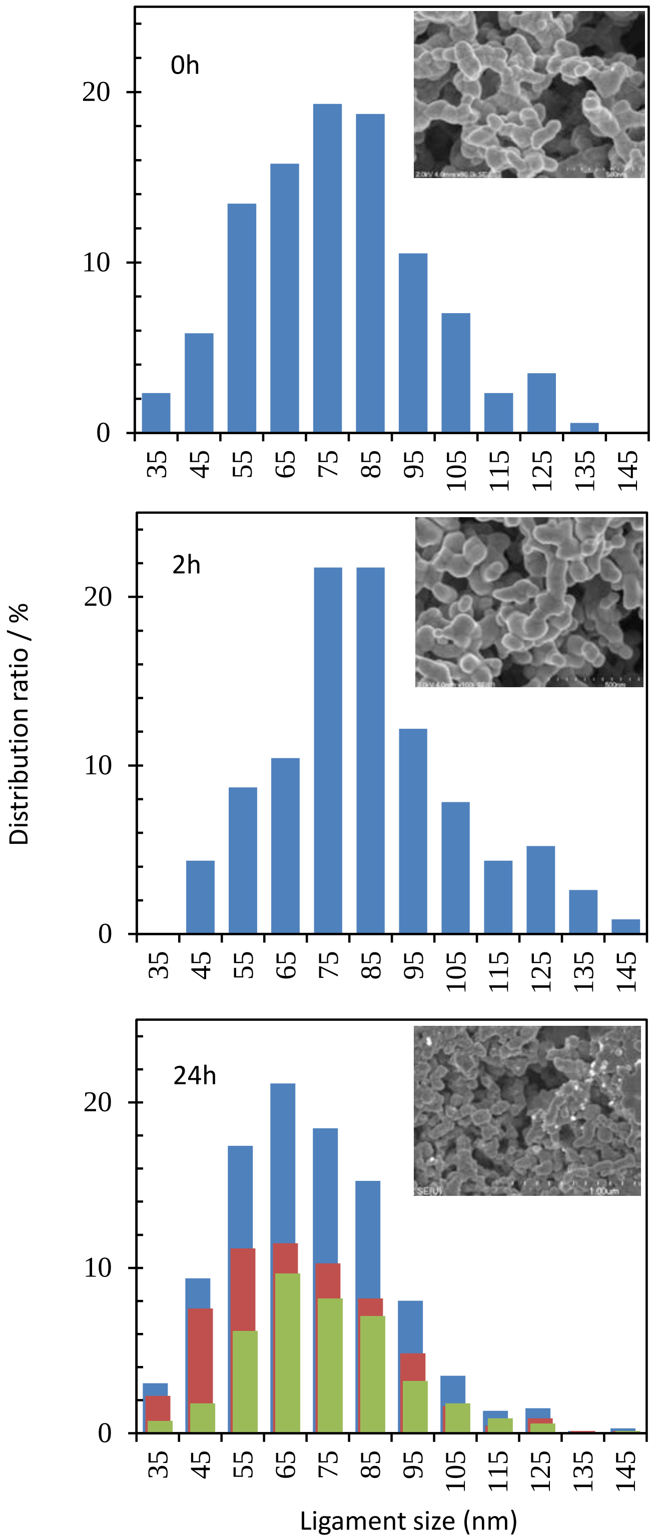

Figure S3. Histogram distribution of ligament size of the dealloyed np-Ag-NPs debris after iodine functionalization at different time. The green, red, and blue bars in the bottom figure represent those in loose-packed (porous) area, close-packed area, and their total average, respectively.
